# Understanding the impact of plasma functionalized MWCNTs on the structure, physicochemical and mechanical properties of PEMA

**DOI:** 10.1038/s41598-025-88246-3

**Published:** 2025-02-08

**Authors:** Omar F. Farag, N. A. M. Eid, Essam M. Abdel‑Fattah

**Affiliations:** 1 Physics Department, Faculty of Science, Zagazig University, Zagazig, 44519 Egypt; 2 Department of Physics, College of Science and Humanities, Prince Sattam Bin Abdulaziz University, P.O. Box 173, 11942 Al Kharj, Saudi Arabia; 3 Plasma Lab, Physics Department, Faculty of Science, Zagazig University, Zagazig, 44519 Egypt

**Keywords:** f-MWCNTs, PEMA, Optical, Thermal stability, Mechanical, AC conductivity, Applied physics, Plasma physics

## Abstract

In this study, plasma functionalized multiwalled carbon nanotubes, f-MWCNTs, were incorporated into a poly(ethyl methacrylate), PEMA, polymer matrix at different wt.% (0.005, 0.01, and 0.02 wt.%) to prepare nanocomposite films using the traditional solution casting method. The XRD, Raman spectroscopy, XPS, TGA, mechanical analysis and UV–Vis spectroscopy techniques were employed to investigate the effects of the wt.% of f-MWCNTs on the structure, spectroscopic and other physiochemical properties of the synthesized films. XRD analysis showed a monotonic change in the PEMA structure upon incorporation of f-MWCNTs at different wt.%. The XPS results showed an increase of oxygen-based functional groups C-O and O-C-O on the PEMA/f-MWCNTs/ composite films compared to pure PEMA. Raman spectroscopy results consistent with the XRD and XPS findings, confirming the homogeneous distribution of f-MWCNTs in the PEMA matrix. Thermal stability of f-MWCNTs/PEMA improved as the f-MWCNTs content increased. Optical studies showed a reduction in the bandgap energy as the f-MWCNTs content increased, accompanied by significant improvements in optical properties such as refractive index (n), extinction coefficient (k), dielectric constants (ε′ and ε″), and optical conductivity (σ_opt_). Mechanical testing revealed enhancements in breaking strength, Young’s modulus, yield stress, and elongation at break with increasing f-MWCNTs concentrations. Furthermore, the AC electrical conductivity of the films also improved, demonstrating better charge transport capabilities. These synergistic enhancements in optical, thermal, mechanical, and electrical properties make PEMA/f-MWCNTs nanocomposites promising candidates for advanced applications, including optoelectronic devices, optical components, and conductive packaging materials.

## Introduction

Polymers incorporating MWCNTs have received increased attention due to their potential technological applications^1^. These composites exhibit improved thermal, mechanical, and electrical properties based on filler dimensions (MWCNTs) and the dispersion ability of the polymer matrix^2,3^. Polymer/MWCNTs composites are used in sensors, electromagnetic interference (EMI) shielding^4^, biomedical devices^5^, energy storage^2,6^, electrochemical^7^ and aerospace applications^8^.

The poly(ethyl methacrylate) (PEMA) exhibits superior chemical resistance as well as excellent thermal and mechanical characteristics. PEMA is primarily serves as a shatter-resistant (lightproof) material. Its low cost and simplicity of processing make it an ideal raw material for the manufacturing sector^9^. Indeed, PEMA has been employed as a component in several applications, including superabsorbent material^10^, thermal energy storage^11^, and drug release material^12^. It has also been reported as a barrier and heat-resistant packaging material^13^. Additionally, PEMA’s biocompatibility makes it suitable for packaging medical devices^13^. Therefore, PEMA was selected as the matrix in our current study.

MWCNTs exhibit excellent thermal durability, high conductivity, an accessible exterior surface, and superior mechanical and optical properties^14,15^. However, due to van der waals forces^16^, MWCNTs tend to aggregate within the polymer matrix, compromising their dispersion and negatively affecting the quality of polymer composite films. To improve the dispersion and compatibility of MWCNTs within the polymer matrix, surface functionalization is essential^17,18^. Surface functionalization involves the introduction of various polar functional groups onto the MWCNTs surface, such as amine (-NH), hydroxyl (-OH), carbonyl C = O, and carboxyl (-COOH)^16,19^. This can be achieved by different techniques including plasma, chemical, electrochemical, and mechanical methods^19–21^. Among these, plasma processing stands out as a clean and dry procedure that generates no chemical waste, time-saving and non-destructive as plasma species interreact only within a few angstroms of the surface^22,23^.

Previous research, such as that by Pourfayaz et al.^24^, Abdel-Fattah et al.^25^, and Zidan et al.^26^, has demonstrated that the functionalization of MWCNTs (f-MWCNTs) can significantly improve the structural, optical, electrical, and thermal properties of polymer-based films. These improvements are primarily attributed to the uniform dispersion and effective interaction between f-MWCNTs and the polymer matrix. In this context, the present study seeks to explore the effect of f-MWCNTs on the physicochemical characteristics of poly(ethyl methacrylate) (PEMA), a polymer that has not been as extensively studied in this regard. A deeper understanding of these interactions could lead to the development of advanced PEMA-based composites with enhanced properties for various applications.

Therefore, the current study aims to investigate the influence of plasma f-MWCNTs with a low loading ratio (0.005%, 0.01, and 0.02 wt%) on the structure, optical, mechanical, electrical, and thermal characteristics of PEMA-based nanocomposites. The use of lower loading ratio of f-MWCNTs aims to investigate the threshold at which f-MWCNTs begin to alter the matrix properties . Our findings demonstrate that successful functionalization of MWCNTs by plasma treatment enhances their distribution in the PEMA matrix, which in turn improves the optical, thermal, mechanical, and electrical properties of the produced films.

## Experimental setup

### Materials

Poly(ethyl methacrylate) (MW = 515,000 g/mol), and acetone were purchased from Sigma-Aldrich, while MWCNTs (~ 30 nm in diameter) were obtained from Hanwha Nanotech, South Korea.

### The plasma surface functionalization of the MWCNTs

The plasma surface functionalization of the MWCNTs was carried out by applying RF dielectric barrier discharge in an argon/nitrogen gas mixture at processing conditions: plasma power 60 W, gas pressure 500 Pa, and treatment time 6 min. The details of the plasma system used for the functionalization of MWCNTs were found in our previous work^16^.

### Preparation of PEMA/f-MWCNTs films

We employed the simple casting technique to fabricate virgin PEMA and PEMA loaded with varying wt.% of f-MWCNTs. In summary, one gram of PEMA was dissolved in 60 mL of acetone and stirred continuously at 50^∘^C for one day, yielding a clear and transparent solution. Certain weights of f-MWCNTs were then added to the solution of PEMA. The concentration of f-MWCNTs in PEMA was (wt.% = 0.005, 0.01, and 0.02). The mixture of PEMA and f-MWCNTs was stirred for one hour before being sonicated (Cole Parmer-CPX 750) for an additional 30 min to achieve a uniform distribution of f-MWCNTs in the PEMA solution. Finally, the homogenous mixes of PEMA/f-MWCNTs were cast on glass plates and air-dried for 24 h. The fabricated film thickness was 30 µm, measured using a digital micrometer.

### PEMA/MWCNTs film characterizations

To investigate the structure and crystallinity of the fabricated films, an X-ray diffraction spectrometer (Rigaku International Corp., Japan) was used, where λ = 1.543 Å, the Bragg’s angle (2θ) at the range of 5–60^◦^, and the tube operating at 30 kV. Also, Micro Raman spectrometer (SENTERRA II, Brucker) at laser λ = 785 nm of power of 1 mW to examine the structure of the films. The PEMA and PEMA/f-MWCNTs films chemical compositions/bonding have been investigated employing XPS (Thermo Scientific™ K Alpha spectrometer) utilizing Al K alpha (1486.6 eV) with a flood gun for charge compensation. C 1s at 284.5 eV was employed for standard calibration. The optical characteristics were examined at room temperature with an ultraviolet–visible spectrophotometer (UV/Vis., V-570 UV/VIS/NIR, JASCO, Japan). The absorbance, transmittance, and reflectance spectrum were measured at a wavelength range of 200–900 nm. The films’ thermal stability was assessed using thermogravimetric analysis (TGA) on (TGA, NETZSCH instruments, Deutschland) under nitrogen flow and heating rate of 10 °C per minute, from 25 to 800 °C. The TGA analysis was conducted using the Netzsch proteus 70 software. The mechanical property analysis was examined at constant strain rate and working temperature of 3.3 × 10^–4^ s^-1^ and 25°C, respectively. By using the tensile testing machine, the mechanical properties were obtained. An average of three testing data were obtained for each composite sample at each processing condition. The ac conductivity, σ_ac_, was measured using an automated programmed RLC meter (FLUKE PM6306). We placed 1.5 cm silver-coated disk samples between two polished brass electrodes to measure the sample bulk resistance.

## Results and discussion

### X‑ray diffraction analysis (XRD)

**Fig. 1:**
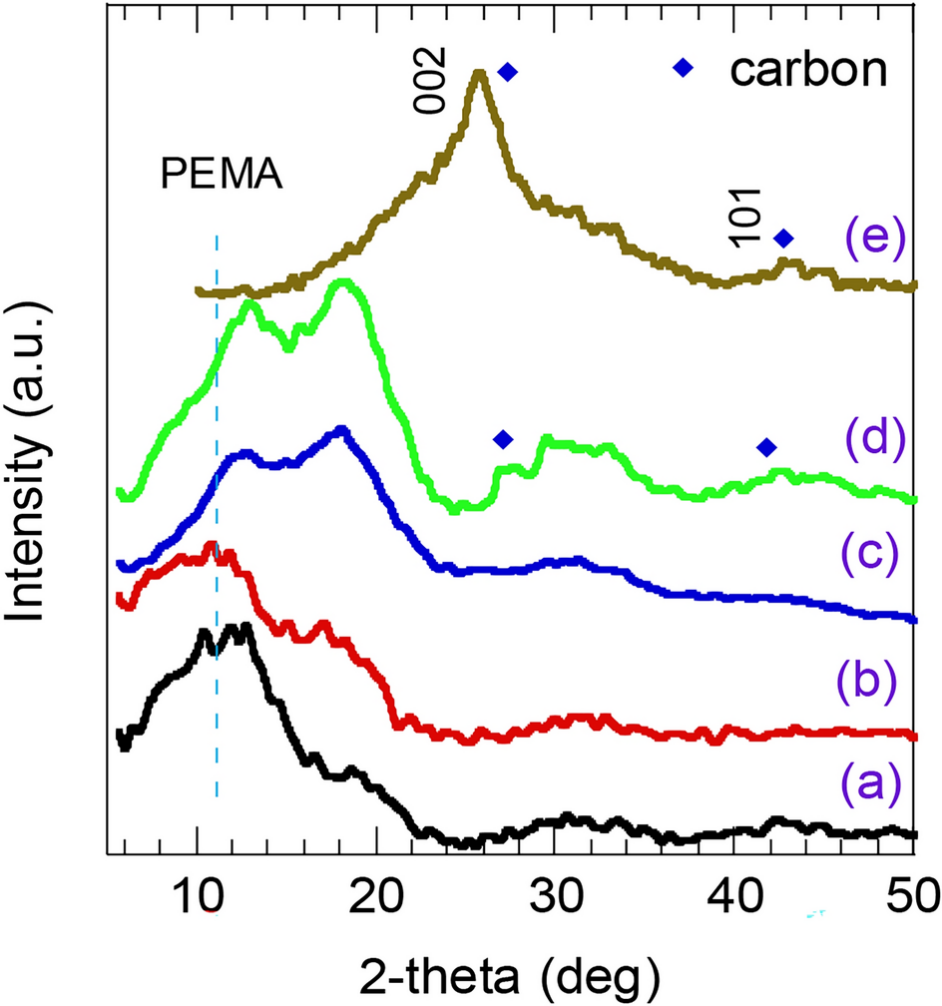
XRD patterns of (**a**) neat PEMA, PEMA loaded with various wt.% of f-MWCNTs (**b**) 0.005%, (**c**) 0.01% and (**d**) 0.02%. (**e**) plasma functionalized-MWCNTs (f-MWCNT).

The XRD spectra of the neat PEMA and PEMA loaded with 0.005, 0.01 and 0.02 wt.% of f-MWCNTs nanocomposite films are shown in Fig. 1. The XRD pattern of f-MWCNTs is also plotted in Fig. 1(e). The obtained spectrum for the neat PEMA demonstrates an amorphous characteristic^27^, with hump centered at 2θ ~ 11^◦^- 14^◦^^9^ as seen in Fig. 1(a). The XRD pattern of PEMA loaded with f-MWCNTs sample (0.005 and 0.01 wt.%) preserves semicrystalline feature of PEMA as shown in Fig. 1(b, c). Yet their XRD peak/hump become more pronounced, and they are shifted to higher 2θ value. The presence of f-MWCNTs within the PEMA matrix might cause internal stress and result in a shift to 2θ value. It was reported that the well-dispersed and aligned nanotubes may raise the X-ray scattering intensity, resulting in a more pronounced peak^28^. This observation implies a good interaction between the f-MWCNTs and PEMA matrix. As the f-MWCNTs wt.% increased to 0.02% in the PEMA films, the PEMA peaks/humps getting more broadening and sharpened. Further, new peaks at 27° and 42° can be seen in Fig. 1(d) that aligned with the 002 and 101 peaks of carbon^18^ as shown in Fig. 1(e) signed to carbon. This is an advantage of plasma functionalization of MWCNTs that enables homogenous distribution of the MWCNTs within the PEMA matrix.

### Raman spectroscopy analysis

**Fig. 2:**
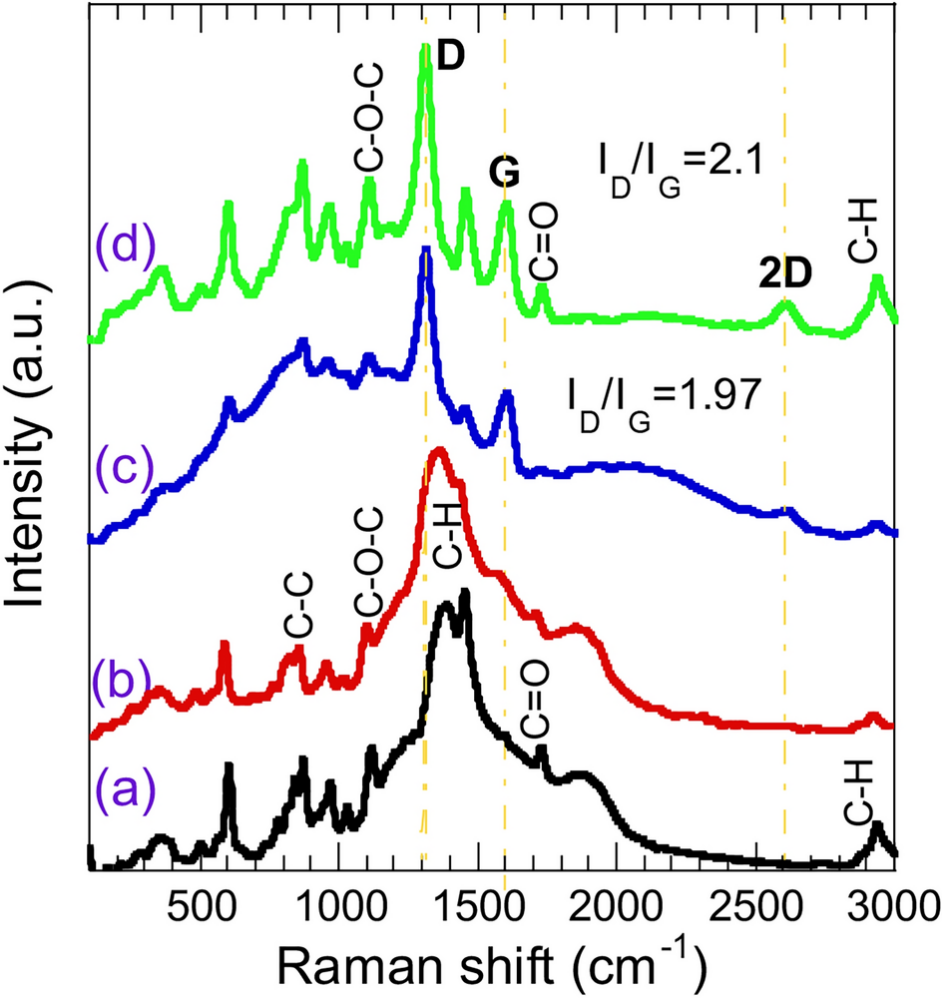
Raman spectra of (**a**) neat PEMA, PEMA loaded with various wt.% of f-MWCNTs (**b**) 0.005%, (**c**) 0.01% and (**d**) 0.02%.

The interaction between the f-MWCNTs and the PEMA matrix was further investigated using Raman spectroscopy, and the results are depicted in Fig. 2. The Raman spectrum of the neat PEMA (Fig. 2(a)) is dominated by a wide, strong peak at 1390–1460 cm^-1^ representing bending vibration of C-H bond in the methyl groups. Other tiny peaks can be seen in Fig. 2(a) at 850 cm^-1^, 1100 cm^-1^, and 1720 cm^-1^ that are corresponding to C–C (PEMA backbone), C–O–C (ester linkage in PEMA) and C = O (carbonyl in the ester group of PEMA) bonds, respectively^29^. The incorporation of f-MWCNTs in the PEMA matrix alters the polymer structure, particularly at higher f-MWCNTs loading of ≥ 0.01 wt.%. The Raman spectrum of the f-MWCNTs (0.005 wt.%) resamples that of neat PEMA, except a slight broadening of the main peak at 1390–1460 cm^-1^. Yet, as the wt.% of f-MWCNTs increased in the PEMA matrix ≥ 0.01, aside from the PEMA Raman modes, new peaks appeared at ~ 1309 cm^-1^, 1602 cm^-1^ and 2600 cm^-1^ which are assigned to *D, G* and 2*D* carbon bands, respectively^30^. These results are consistent with the XRD results of f-MWCNTs/PEMA nanocomposite (Fig. 1). The *D* peak represents disorder carbon structure, the *G* band correspond to the well-ordered carbon structure and the 2D peak is overtone of the *D*-band. The I_D_/I_G_ peak ratio reflects the degree of disorder in carbon materials^31^. A higher I_D_/I_G_ ratio implies a greater degree of disorder in the carbon structure. As is evident, the I_D_/I_G_ ratio increases when the f-MWCNTs loading ratio increases to 0.02%. A similar observation was reported for MWCNTs incorporated PMMA^32^.

### XPS analysis

**Fig. 3:**
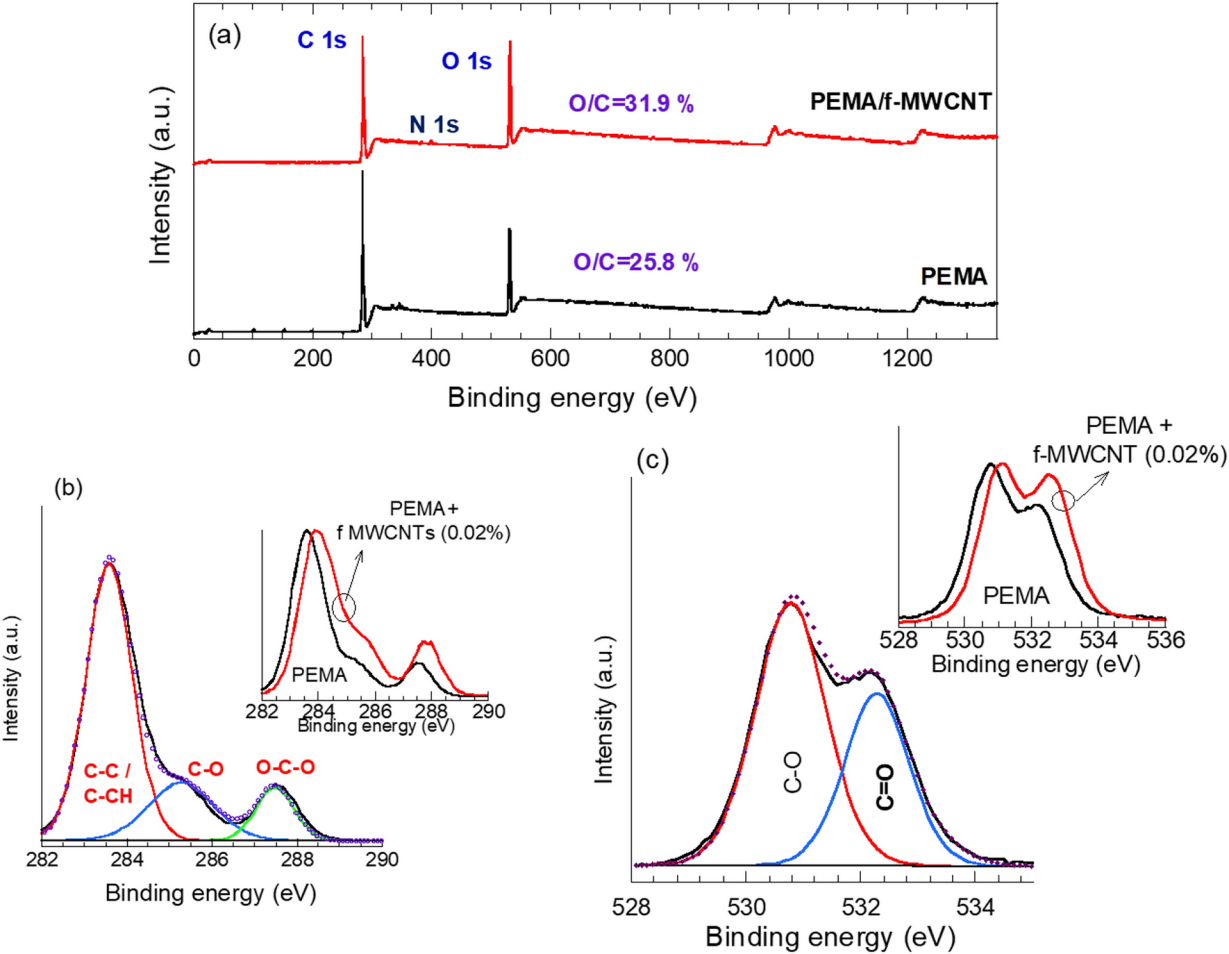
(**a**) Survey spectra of pristine PEMA and PEMA/f-MWCNTs samples, (**b**) C 1s and (**c**) O 1s of spectra of pristine PEMA sample.

We further investigate the chemical compositions of the films using the XPS technique. Figure 3(a) shows the survey spectra of the pure PEMA and PEMA/f-MWCNTs composite films. The survey spectrum of pure PEMA polymer dominated with two strong peaks at 283.9 eV and at 531.5 eV. The peak at 283.9 eV is assigned to carbon C 1s while the peak 531.5 eV corresponds to oxygen O 1s. No other unknown peaks were observed, confirming the purity of the synthesized PEMA film. The O 1s to C 1s ratio in pure PEMA is 25.8%. Similarly, the survey spectrum of the PEMA/f-MWCNTs composite film revealed strong peaks assigned to C 1s and O 1s, but with an increased O 1s content. The O 1s/C 1s ratio is 31.9%. The increase in the O 1s/ C 1s ratio in the PEMA/f-MWCNTs composite film compared to pure PEMA is attributed to the successful functionalization of the plasma treated MWCNT. Further, a small peak at 400 eV, assigned to nitrogen N 1s can be seen in the survey spectrum of PEMA/f-MWCNTs composite film.

To identify the various functional groups associated with carbon in the examined films, Fig. 3(b) shows the deconvolution of the C 1s spectrum. Three sub-peaks characteristic of PEMA polymer^33^. The C–C or C–CH bonds at 283.8 eV, C-O at 285.4 eV, and O-C-O species at 287.5 eV. The incorporation of f-MWCNTs into the PEMA matrix enhanced the oxygen-based functional group C-O and O-C-O as seen in the inset of Fig. 3(b). These results confirm the chemical interaction between PEMA and the incorporated f-MWCNTs. The O1s spectrum of the pristine PEMA is presented in Fig. 3(c), where it is fitted into two sup-peaks; one at 530.8 eV corresponding to C-O and another at 532.5 eV, assigned to carbonyl oxygen group, C = O. From the inset of Fig. 3 (c), the incorporation of f-MWCNTs into PEMA enhanced the carbonyl group (C = O) in the PEMA/f-MWCNTs composite films, which aligns with the Raman results. Furthermore, shifts to higher B.E. of the C 1s and O 1s spectra (insets of Fig. 3(b, c)) in the PEMA/f-MWCNT composite films are observed. These shifts are likely due to improved electrical conductivity of PEMA upon the incorporation of f-MWCNTs.

### Thermal analysis

**Fig. 4:**
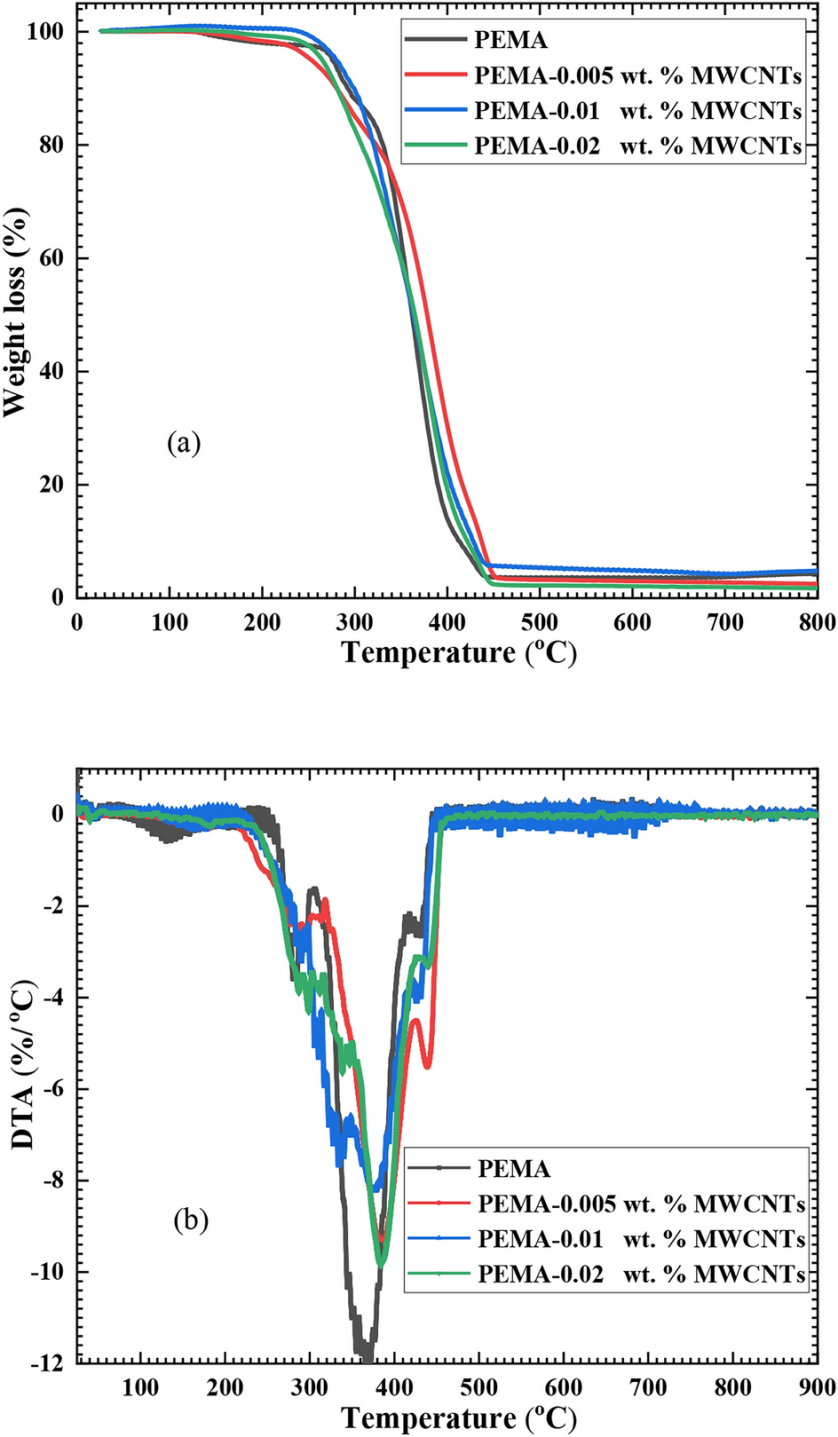
TGA (**a**) and DTGA (**b**) curves for pristine PEMA and PEMA/f-MWCNTs samples.

The TGA and its 1^st^ derivative DTA for the pristine PEMA, as well as PEMA loaded with various wt.% of f-MWCNTs nanocomposites films, are depicted in Fig. 4. PEMA degrades in three steps, with the initial stage occurring above 110°C owing to loss of moisture and solvent evaporation^34^. In this stage, the pristine PEMA loses about 1.86% of its initial weight. The next stage takes place at 270 to 330°C, while the final stage takes place at 330 to 440°C. Structural deformation takes place during the second stage of degradation since the pristine PEMA loses about 11.34% of its initial weight. In the final stage, the pristine PEMA loses about 81.4% of its initial weight due to the partly degraded PEMA oxidizing. Thermal degradation finished at 440°C, and the residual weight was 5%. By comparing the weight loss profiles of PEMA loaded with (0.005, 0.01, and 0.02 wt.%) of plasma-f-MWCNTs with that of the pristine PEMA, it was found to be identical over the studied temperature range. Also, the addition of f-MWCNTs to the PEMA matrix was found to enhance its thermal durability, causing the degradation temperatures to rise. Furthermore, as revealed in Fig. 4(b), the f-MWCNTs raise the PEMA composites’ maximum decomposition temperature (T_m_) slightly. The T_m_ was 367^∘^C for the pristine PEMA and increased to 378^∘^C, 384^∘^C, and 376^∘^C for PEMA/f-MWCNTs samples with loading ratios of 0.005, 0.01, and 0.02 wt.%, respectively. This enhancement can be attributed to slower degradation of polymer chains near nanotubes, resulting in higher polymer decomposition temperatures^35^. The improvement in PEMA thermal stability by loading f-MWCNTs can be ascribed to the strong interactions between f-MWCNT and the chains of PEMA, which restrict the motion of PEMA main chains. The higher thermal conductivity of f-MWCNTs promotes the dissipation of heat throughout the composite^36^. Abdel-Fattah et al.^25^ found that loading PVC films with 0.02 wt.% of f-MWCNTs enhanced their thermal stability and increased the temperature at maximum weight loss by 20.4^∘^C.

### Optical properties analysis

**Fig. 5:**
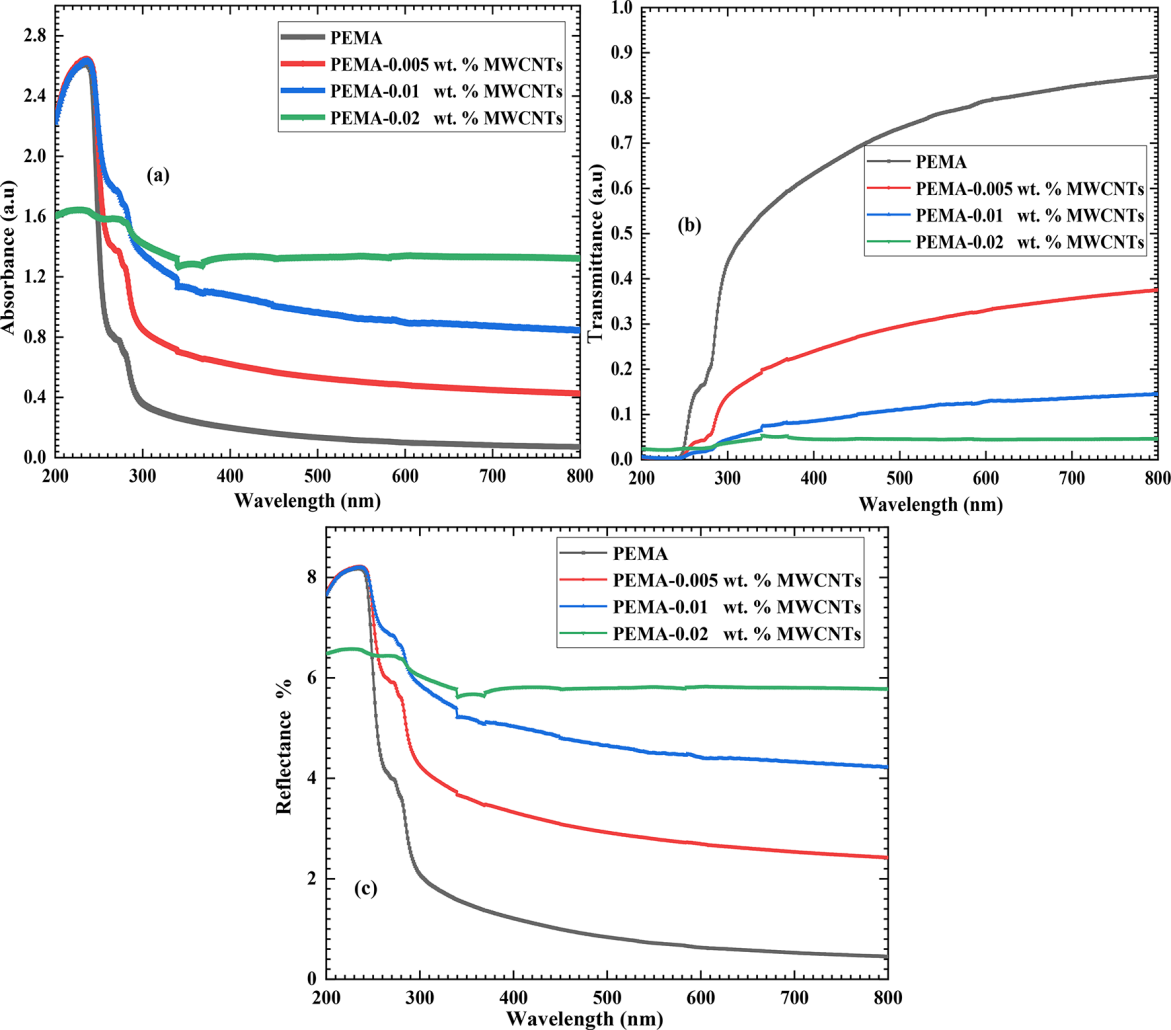
The UV/VIS optical (**a**) absorption, (**b**) transmittance, and (**c**) reflectance spectra of PEMA and PEMA/f-MWCNTs composite films.

The UV/VIS optical absorption, transmittance, and reflectance spectra of PEMA and PEMA/f-MWCNTs composite films are shown in Fig. 5. In the case of pure PEMA, a strong absorption peak is observed in the UV absorption region at 230 nm, along with a shoulder-like band at 275 nm. These features are attributed to the π -π* transition in C = O^37^. The UV–VIS absorption spectra of PEMA/f-MWCNTs nanocomposites reveal a strong absorption peak and a shoulder-like band in the UV region, similar to pristine PEMA, without the appearance of new peaks. The inclusion of f-MWCNTs into PEMA is evidenced by the observed shift of the absorption edge of PEMA/f-MWCNTs composite films towards longer wavelengths, which indicates a significant decrease in band gap energy in the PEMA/f-MWCNTs composites. Additionally, absorption increases with increasing f-MWCNT loading ratios in the PEMA matrix, except for the PEMA/0.02% f-MWCNTs sample in the 200–290 nm wavelength range. The increased absorption of PEMA/f-MWCNTs composites compared to pristine PEMA is probably attributed to the high absorption characteristic of MWCNTs^18,25^.

Figure 5(b) depicts the transmittance T(λ) of the pristine PEMA and PEMA/f-MWCNT nanocomposite films. The value of T(λ) in the λ < 245 nm region is ~ 1%, which is attributed to the strong UV absorbance of the pristine PEMA film. As λ increases from 245 to 325 nm, a significant increase in transmittance from ~ 1% to 50% is observed. It further increases from 50 to 85% in the wavelength range of 325 to 800 nm. Additionally, all PEMA/f-MWCNTs films exhibit T(λ) values lower than 2% in the λ < 245 nm region. Moreover, the inclusion of f-MECNTs into the PEMA matrix reduces the film transmittance in the 300—800 nm range. T(λ) at 600 nm (T_600_) is dramatically reduced from 80 to 32%, 13%, and 4% for f-MECNTs lading ratios of 0, 0.005, 0.01, and 0.02 wt.%, respectively. This suggests that PEMA with plasma-f-MWCNTs, PEMA/f-MWCNTs films can prevent/or reduce the transmission of light through them, making them an appropriate material for applications in food packaging. R(λ) spectra revealed a reverse curve-tendency behavior compared with T(λ). The reflectance R(λ) for the pristine PEMA and PEMA/f-MWCNTs exhibits a similar wavelength-dependent behavior. The inclusion of f-MWCNTs into PEMA film enhances the reflectance across the entire spectrum, as shown in Fig. 5(c). Our findings are consistent with those reported previously by Helal et al.^38^.

**Fig. 6:**
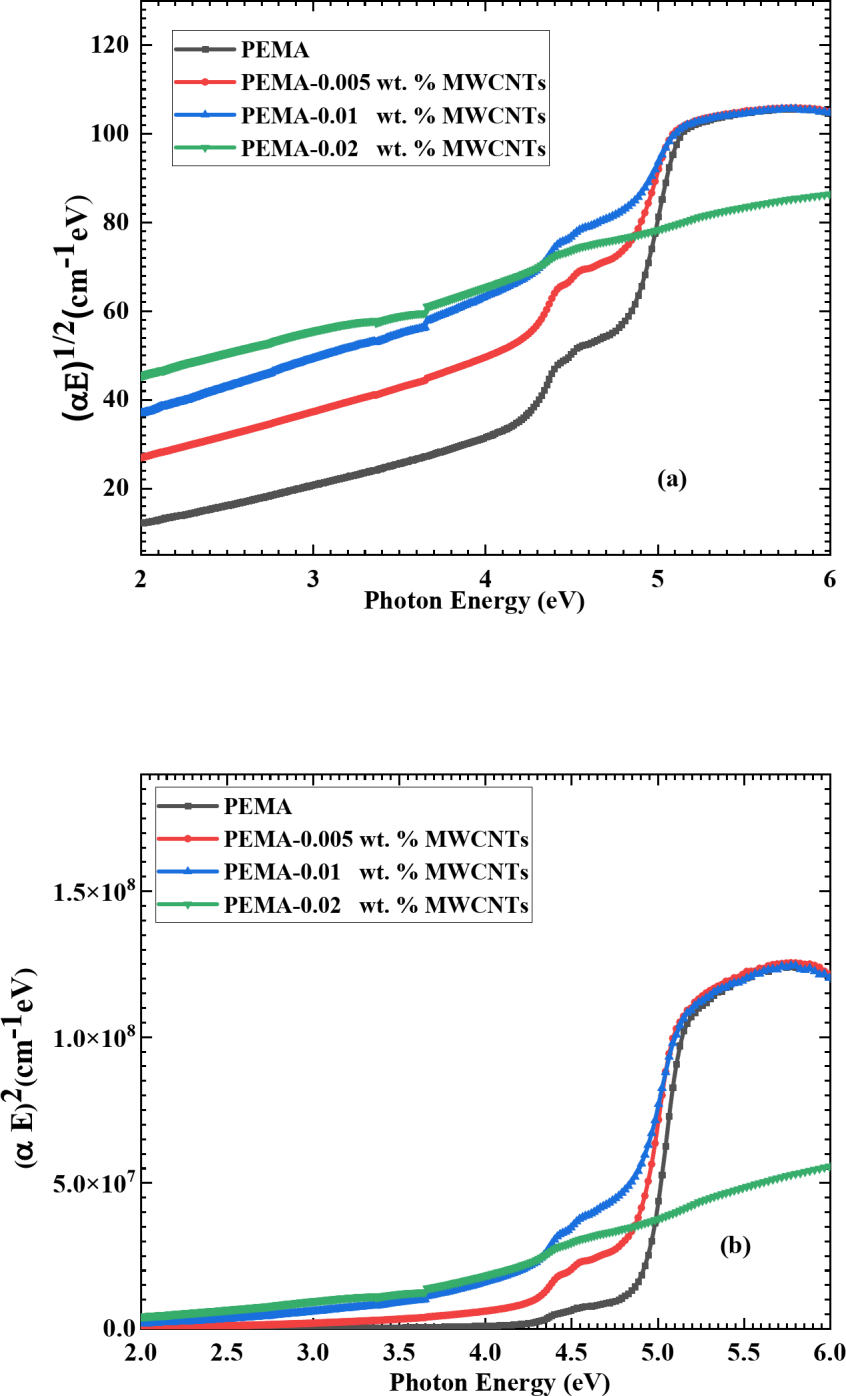
Variation of [α E]^0.5^ (**a**) and [α E]^2^ (**b**) with the energy of photon of neat PEMA and PEMA loaded with various wt.% of f-MWCNTs.

The plots of (αhv)^1/2^ and (αhν)^2^ versus the energy of the photon, (hν) depicted in Fig. 6(a, b), follow the Tauc equation: (αhν) = A(hν-E_g_)^x^, where α is the coefficient of absorbance, E_g_ is the optical energy gap, *x* is an exponent that describes the transition procedure, with values of 0.5 and 2 for direct and indirect transitions, respectively, and A is a constant^39^. The values of indirect optical energy gap and direct optical energy gap were calculated directly from the plots of (αhν)^0.5^ and (αhν)^2^ vs hν, respectively, by the intercept of the linear section of the curves in Fig. 6 to cut the x-axis (or the photon energy-axis) at zero of (αhν)^0.5^ and (αhν)^2^, respectively, and their values are listed in Table 1. As shown in Table 1, the values of indirect and direct E_g_ for PEMA films go down as the f-MWCNTs loading ratio in the PEMA matrix goes up. The indirect and direct E_g_ values for the pristine PEMA are 4.55 and 4.9 eV, respectively, whereas they fall to 2.97 and 3.49 eV for PEMA/f-MWCNTs film loaded with 0.02 wt.% of f-MWCNTs. The loaded f-MWCNTs facilitate the charge transfer between the nanotubes and the polymer matrix, which modify the composite electronic structure^39^. Moreover, f-MWCNTs creates localized states in the band structure of PEMA acts as additional energy levels within the band gap^24,40^. All these factors collectively contribute to the observed decrees in the band gap when f-MWCNTs are loaded into a PEMA matrix. The E_g_ values obtained for the samples under investigation are consistent with prior studies^18,38^.

**Table 1:** Direct and indirect energy gap values for pristine PEMA and PEMA doped with different wt.% of f-MWCNTs.

Samples	Direct energy gap (eV)	Indirect energy gap (eV)
PEMA	4.90	4.55
PEMA-0.005 wt. % MWCNTs	4.80	4.18
PEMA-0.01 wt. % MWCNTs	4.77	3.66
PEMA-0.02 wt. % MWCNTs	3.49	2.97

**Fig. 7:**
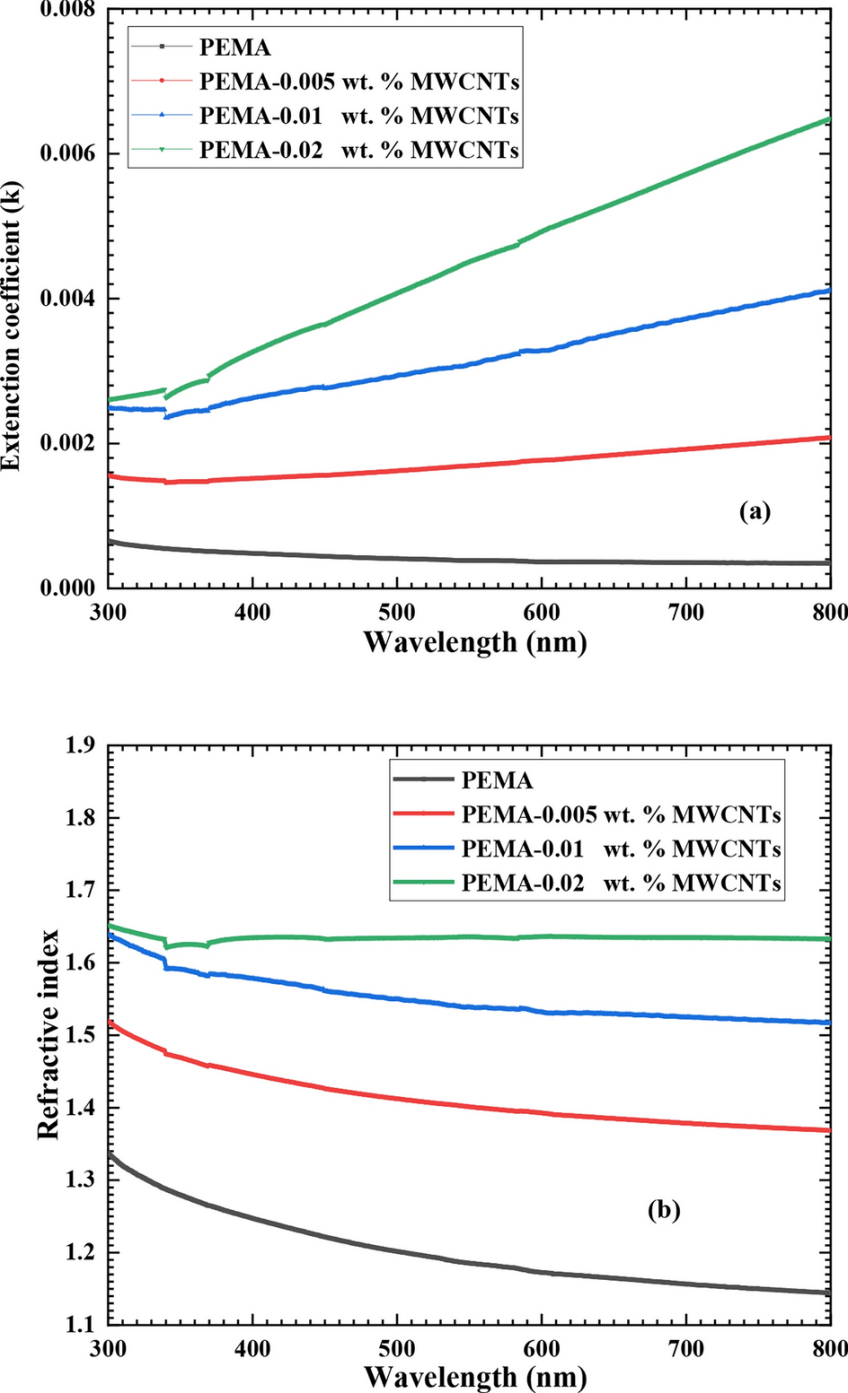
The extinction coefficient (k) (**a**) and refractive index (n) (**b**) against the wavelength for neat PEMA and PEMA loaded with various wt.% of f-MWCNTs.

The extinction coefficient (k) measures the dissipation rate of the electromagnetic wave in the dielectric medium. It was estimated from [*k* = $\alpha \lambda /4\pi ]$, where α is the absorption index calculated from absorption spectra^26^. The refractive index (n) was estimated from the measured value of reflectance [R(λ)] and the obtained values of (*k*) according to Fresnel’s formula^41^. Figure 7 plots the extinction coefficient (k) and refractive index (n) against wavelength for the pristine PEMA and PEMA/f-MWCNTs films. It has been shown that both (*k* & n) of the pristine PEMA film nearly decline with increasing the wavelength in the wavelength range 300–800 nm. While for PEMA/f-MWCNTs, *k* takes an increasing trend, and n takes decreasing trend with increasing the wavelength. While for PEMA/f-MWCNTs, k increases, and n decreases as the wavelength increases. Also, at any λ in the studied range (300–800 nm), the values of both (*k* and n) of the PEMA/f-MWCNTS nanocomposite films are greater than those of the pristine PEMA. Furthermore, both (*k* and n) of the PEMA/f-MWCNTs nanocomposite films increase when the f-MWCNTs wt.% increases from 0 to 0.02%. The enhancement in *k* values of the PEMA with the inclusion of f-MWCNTs into PEMA matrix is due to high absorption coefficient of MWCNTs, which increases its optical absorption. Introducing f-MWCNTs may cause a structural alteration in the PEMA matrix, which leads to the observed increase in (n) values^42^. Also, introducing f-MWCNTs into the PEMA matrix increases the packing density of the film, which in turn increases the value of n^18^. Similar findings were achieved when the amounts of MWCNTs wt.% increased from 0 to 4 wt.% in PEO/PVA nanocomposite films; the refractive index increased from 1.5 to 1.7 at λ = 700 nm, respectively^43^. Also, El-Gamal et al.^44^ observed that including MWCNTs into the PAAM/CMC blend enhances the (n) value in the region of UV from 1.44 to 1.67 as the loading ratio increases from 0.1 to 2 wt.%.

**Fig. 8:**
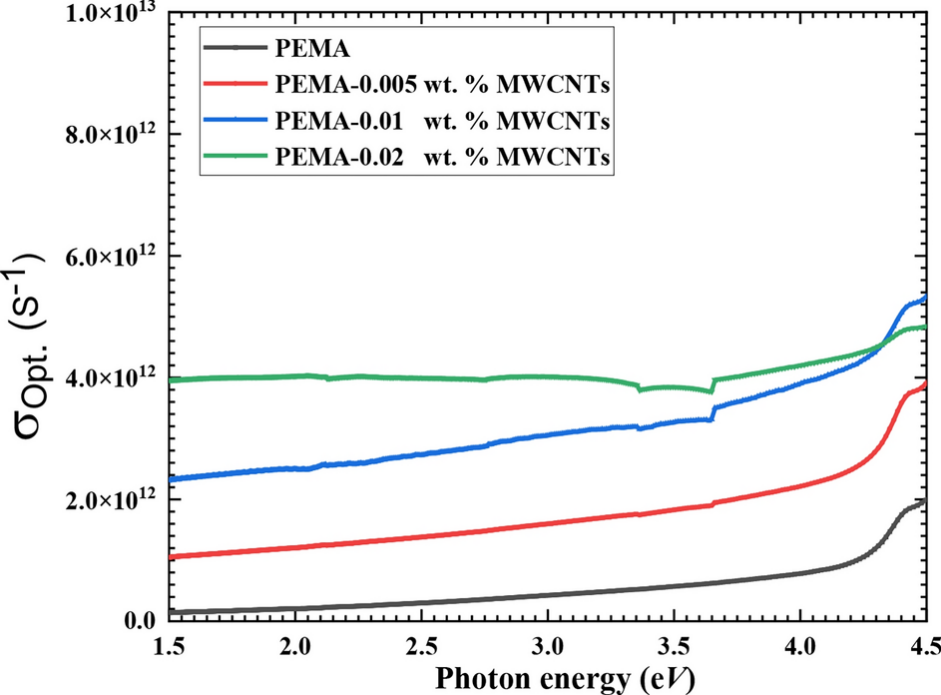
The optical conductivity versus the photon energy for neat PEMA and PEMA loaded with various wt.% of f-MWCNTs.

Optical conductivity (σ_opt._) is a crucial metric for understanding the charge transfer between f-MWCNTs and PEMA polymer. It is the material’s reply to the diffusion of charge carriers generated by the incoming photon’s energy. The values of optical absorption coefficient (α) and refractive index (n) are used to calculate σ_opt._ for the pristine PEMA and PEMA/f-MWCNTs films utilizing the formula: σ_opt._ = nαc ⁄ 4π, where c is the speed of light^45^. According to Fig. 8, σ_opt._ behaves similarly to optical absorption. At any incident photon energy lower than 4.2 eV, the σ_opt._ of the PEMA/f-MWCNTs nanocomposite films is greater than that of the pristine PEMA polymeric matrix. For instance, at hν = 4 eV, the σ_opt._ for the pristine PEMA was 7.92 × 10^11^ s^-1^, whereas it rose to 7.4 × 10^12^ s^-1^ for the PEMA/f-MWCNTs film loaded with 0.02 wt.% of f-MWCNTs. Our findings agree with those published in the literature^18,38,46^. This enhancement in the PEMA σ_opt._ values by loading f-MWCNTs could be ascribed to the formed extra energy levels in the forbidden band gap, which facilitates electron transition from one state to another^38^. Hence, the observed increase in optical conductivity and the reduction in E_g_.

Moreover, the permittivity, also known as the dielectric function (ε*), is a complex quantity that connects the refractive index (n) and extinction coefficient (*k*) as follows^47^:

$$\varepsilon^{*} = \varepsilon_{r} - i\varepsilon_{i}$$

with ${\varepsilon }_{r}{{=n}^{2}-{k}^{2}}$ and ${\varepsilon }_{i}=2nk$, where ɛ_r_ and ε_i_ are the dielectric function’s real and imaginary components, respectively. The (ε_r_) represents the energy density levels that cause scattering in the sample and impact the light speed. The (ε_i_) reveals energy absorbed due to dipole motion and follows the behaviour of (*K*).

**Fig. 9:**
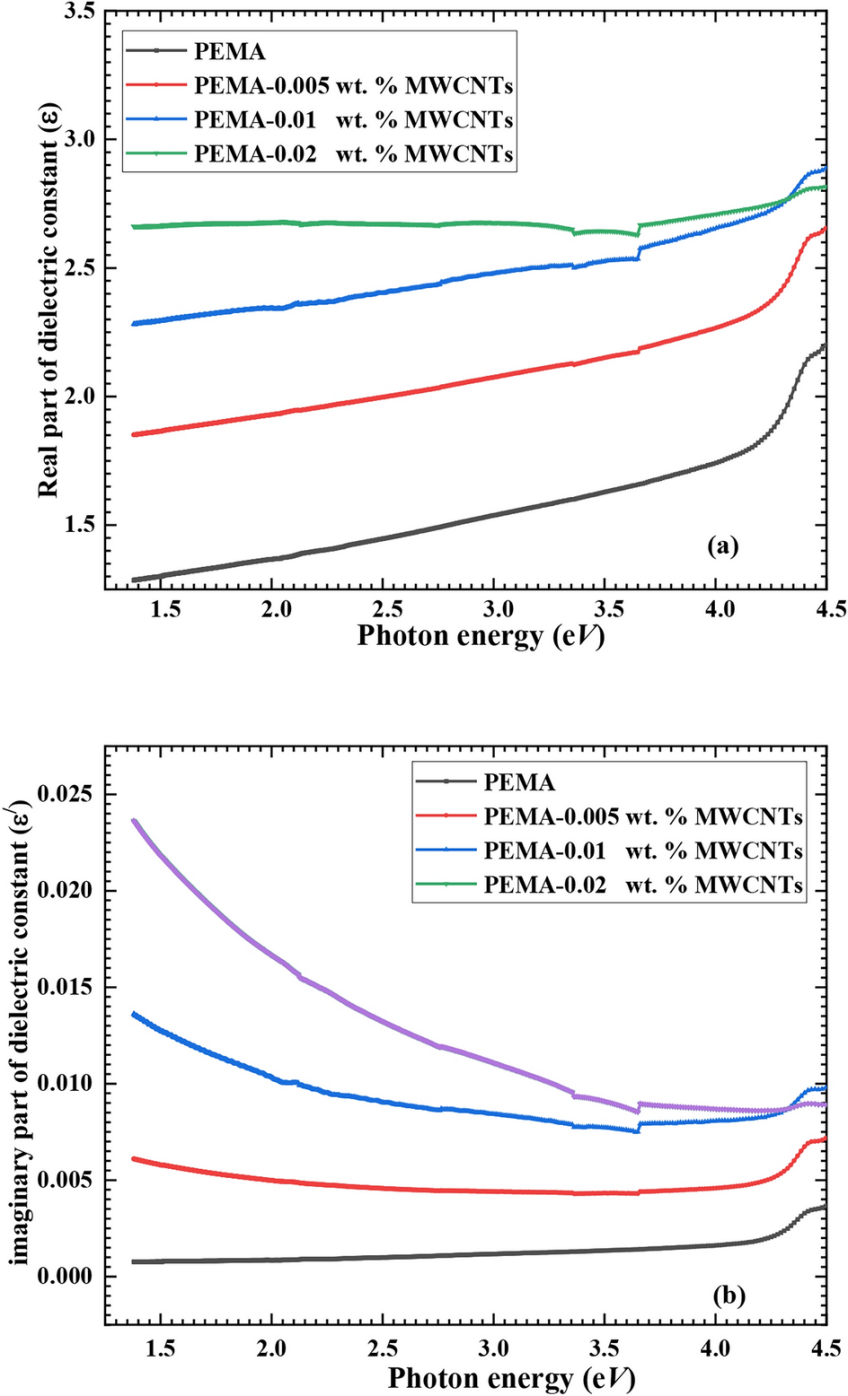
(**a**) The real and (**b**) imaginary parts of dielectric constant for neat PEMA and PEMA loaded with various wt.% of f-MWCNTs.

Figures 9(a, b) illustrate the real and imaginary components of the dielectric constant of the pristine PEMA and different wt.% of f-MWCNTs filled PEMA polymeric films against the energy of photons (hv). It was observed that both ε_r_ and ε_i_ of PEMA/f-MWCNTs are higher than that of the pristine PEMA and rise as the wt.% of the f-MWCNTs in the film increases. These findings can be stated in terms of the nanocomposite films’ density of states (DOS): As the loading ratios of f-MWCNTs rise, greater numbers of energy density states are present, resulting in a rise in polarization, which leads to an enhancement in the real part of the dielectric constant of the nanocomposite films. While the rise in the imaginary component of the optical dielectric constant as f-MWCNTS wt.% grows is mostly due to fluctuations in dipole motion^48^. Our findings agree with those published in the literature^26,38,48^.

### Mechanical properties

**Fig. 10:**
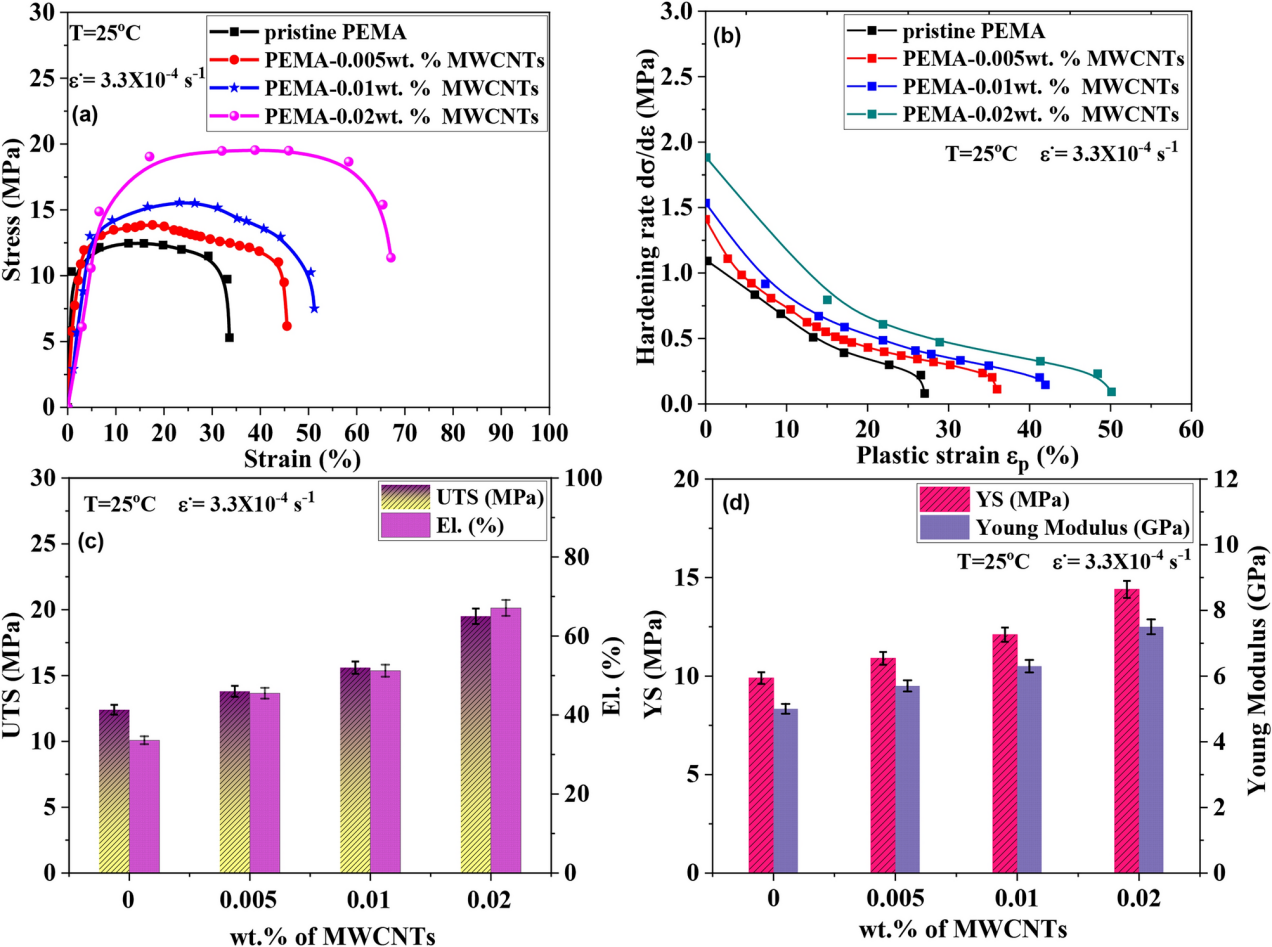
Mechanical behavior of pristine PEMA and PEMA doped with different wt.% of f-MWCNTs composite samples at a strain rate of 3.3 × 10^–4^ s^-1^ and 25 °C. (**a**) Tensile stress–strain curves tested, (**b**) Strain-hardening rate as a function of strain, and (**c** and **d**) corresponding histogram of ultimate tensile stress UTS, elongation, yield stress YS, and Young Modulus.

The stress–strain (σ-ε) tests were conducted to evaluate the impact of f-MWCNTs on the mechanical properties of the PEMA matrix. Figure 10(a) illustrates the σ-ε curves for the fabricated samples at a strain rate of 3.3 × 10^–4^ s^-1^ and T = 25°C. Additionally, vital mechanical properties, including Ultimate Tensile Strength (UTS), Young’s Modulus (E), Yield Stress (YS), and Elongation (El. %), were calculated and are presented in Table 2 and summarized in the corresponding histogram Fig. 10(c and d). The results show that the tensile strength and ductility of composites increase with increasing f-MWCNTs concentrations from 0.005 wt% to 0.02 wt %. Notably, the Pristine PEMA polymer matrix exhibits the lowest UTS (12.4 MPa), E (5 GPa), YS (9.9 MPa) and elongation of 33.6%. However, the UTS increases from 12.4 to 19.5 MPa with an increase of ~ 57.25% in UTS when the addition of f-MWCNTs rises to 0.02 wt%. Furthermore, the YS also increases from 9.9 to 14.4 MPa. Likewise, Young modulus E increases from 5 to 7.5 GPa. Meanwhile, the elongation increases from 33.6 to 67.1% (~112%), which is approximately twice that assessed for the pristine. The enhancement in strength and ductility is attributed to the more robust reinforcement effect of f-MWCNTs when combined with the PEMA matrix, resulting in improved performance in general^49,50^. The considerable improvements in tensile strength and Young’s modulus are thought to be mostly attributable to excellent f-MWCNT dispersion and effective stress transport from the PEMA matrix to f-MWCNTs via interfacial bonding^51^, as confirmed from XRD, XPS, and Ramman spectroscopy outcomes.

Table 2 also revealed that the UTS and Young modulus of low wt.% MWCNTs to PEMA matrix are comparable to several recent works on low and high-wt.% MWCNTs to PVA/SA blend^49^, although the strength of PEMA-x wt. %MWCMTs is higher than most of them.

To demonstrate the flow behavior and strain-hardening response of four composite samples, the strain-hardening rate (dσ/dε) is plotted against plastic strain (ε_p_) in Fig. 10(b) It is seen that the PEMA/0.02wt.% f-MWCNTS sample exhibits the highest strain-hardening rate of 1.9 MPa at ε_p_ = 0 throughout the tensile behavior, while the pristine specimen shows the lowest one of 1.1 MPa. This implies that a higher hardening response may provide greater resistance against localization necking, thereby enhancing the uniform strain and homogenizing the flow behavior.

**Table 2:** Mechanical property data for pristine PEMA and PEMA doped with different wt% of f-MWCNTs at ε• = 3.3 × 10^−4^ s^−1^ and T = 25 °C.

Samples	UTS (MPa)	YS (MPa)	Young modulus (GPa)	El (%)
PEMA	12.4	9.9	5	33.6
PEMA-0.005wt. % MWCNTs	13.8	10.9	5.7	45.5
PEMA-0.01wt. % MWCNTs	15.6	12.1	6.3	51.2
PEMA-0.02wt. % MWCNTs	19.5	14.4	7.5	67.1

### AC electrical conductivity

The ac conductivity (σ_ac_) of pristine PEMA and PEMA/f-MWCNTs nanocomposite films has been estimated utilizing the following formula^52^:

$${{\sigma }_{ac}=l/RA }$$

where R, *l*, and A are the sample bulk resistance, thickness, and cross-section area, respectively.

**Fig. 11:**
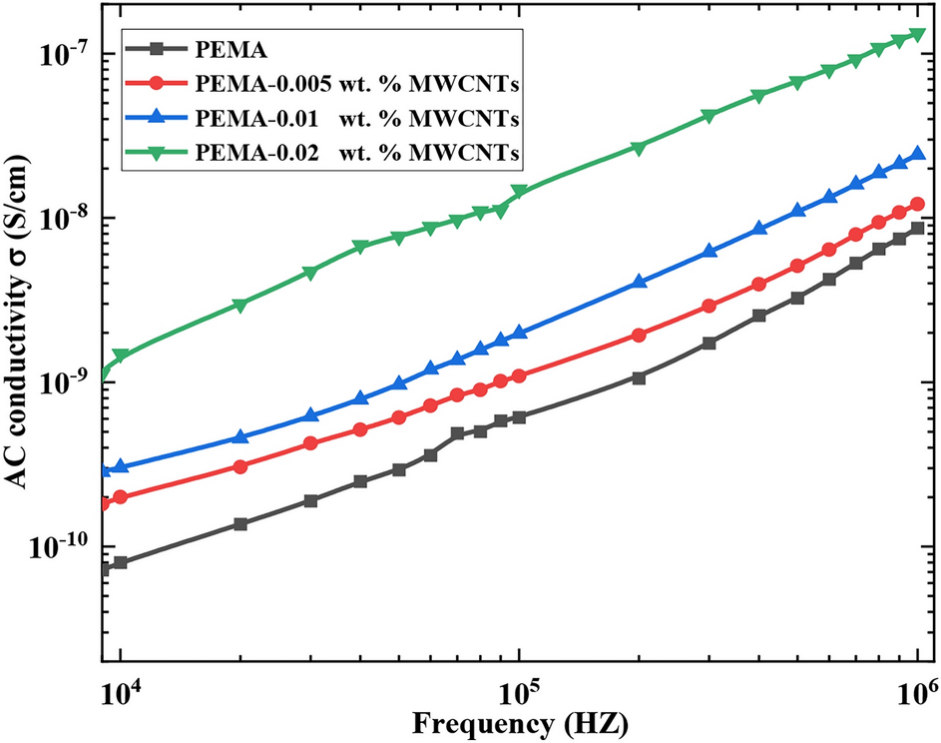
The AC conductivity against the applied frequency for neat PEMA and PEMA loaded with various wt.% of f-MWCNTs.

Figure 11 depicts graphs of the variation in AC conductivity against frequency for the pristine PEMA and PEMA/f-MWCNTs samples at T = 25°C. The values of (σ_ac_) rise with frequency for the pristine PEMA as well as PEMA/f-MWCNTs samples. Moreover, loading PEMA with f-MWCNTs improves σ_ac_. For instance, at frequency 1 MHz, the σ_ac_ for the pristine PEMA was 8.83 × 10^–9^ S/cm, while it was 1.236 × 10^–8^ S/cm, 2.46 × 10^–8^ S/cm, and 1.41 × 10^–7^ for PEMA filled with (0.005, 0.01, and 0.02 wt.%) f-MWCNTs, respectively. Inclusion of 0.02 wt.% f-MWCNTs into the PEMA matrix increases σ_ac_ nearly by one order. The rise in σ_ac_ for the PEMA/f-MWCNTs sample may indicate that the f-MWCNTs, which were conducting additives, created a conducting network on the insulating PEMA matrix. Thus, f-MWCNTs provide a conductive network within the PEMA matrix, permitting the movement of charge carriers. This network reduces the distance that charge carriers need to travel, thereby increasing the overall conductivity^18^.

## Conclusion

PEMA/Plasma-f-MWCNTs films containing (0, 0.005, 0.01, and 0.02 wt.%) were produced using a simple casting method. The impact of f-MWCNTs on chemical structure, thermal, optical, mechanical, and electrical properties was examined. The functionalization and the interaction of MWCNTs with the PEMA matrix were confirmed by XRD, Raman spectroscopy, and XPS techniques. The X-ray evaluation revealed that no significant peaks identifying MWCNTs were found in the nanocomposite containing 0.005 and 0.01 wt.%, but new peaks at 27° and 42° were observed for the composite containing 0.02 wt.% that aligned with the (002) and (101) peaks of carbon. The Raman spectroscopy and XPS analysis showed that the incorporation of plasma-f-MWCNTs in the PEMA enhanced the content of carbonyl groups in the PEMA/f-MWCNTs composite films. The TGA and its 1^st^ DTA revealed an enhancement in the thermal stability of PEMA films with loading f-MWCNTs. When compared to the PEMA matrix, composites with a loading ratio of 0.01 wt.% of f-MWCNTs had a 17°C higher maximum decomposition temperature. As the f-MWCNT wt.% increased, the direct and indirect energy gaps were decreased. Also, the film transmittance was reduced, making them appropriate material for applications in food packaging. Optical parameters, including the index of refraction (n), extinction coefficient (*k*), optical conductivity, and dielectric constants (ε_r_ and ε_i_), were thoroughly studied. Loading PEMA with f-MWCNTs improves the mechanical properties of the fabricated samples. For the composite loaded with 0.02wt.% of f-MWCNTs, the ultimate tensile strength (UTS), YS, and Young modulus (E) increased by 57.3%, 45.5%, and 50% compared to the neat PEMA, reaching 19.5, 14.4 MPa, and 7.5 GPa, respectively. Furthermore, the elongation was doubled. The AC conductivity of the fabricated nanocomposites was enhanced by loading f-MWCNTs.

## Data Availability

All data generated or analyzed during this study are included in this published article.
